# A Short-Term Ketogenic Diet Impairs Markers of Bone Health in Response to Exercise

**DOI:** 10.3389/fendo.2019.00880

**Published:** 2020-01-21

**Authors:** Ida A. Heikura, Louise M. Burke, John A. Hawley, Megan L. Ross, Laura Garvican-Lewis, Avish P. Sharma, Alannah K. A. McKay, Jill J. Leckey, Marijke Welvaert, Lauren McCall, Kathryn E. Ackerman

**Affiliations:** ^1^Australian Institute of Sport, Canberra, ACT, Australia; ^2^Exercise and Nutrition Research Program, Mary MacKillop Institute for Health Research, Australian Catholic University, Melbourne, VIC, Australia; ^3^Griffith Sports Physiology and Performance, School of Allied Health Sciences, Griffith University, Gold Coast, QLD, Australia; ^4^School of Human Sciences (Exercise and Sport Science), The University of Western Australia, Crawley, WA, Australia; ^5^University of Canberra Research Institute for Sport and Exercise, Canberra, ACT, Australia; ^6^Statistical Consulting Unit, Australian National University, Canberra, ACT, Australia; ^7^Division of Sports Medicine, Boston Children's Hospital, Boston, MA, United States; ^8^Neuroendocrine Unit, Massachusetts General Hospital and Harvard Medical School, Boston, MA, United States

**Keywords:** ketogenic diet, bone health, exercise, nutrition, endurance athletes

## Abstract

**Objectives:** To investigate diet-exercise interactions related to bone markers in elite endurance athletes after a 3.5-week ketogenic low-carbohydrate, high-fat (LCHF) diet and subsequent restoration of carbohydrate (CHO) feeding.

**Methods:** World-class race walkers (25 male, 5 female) completed 3.5-weeks of energy-matched (220 kJ·kg·d^−1^) high CHO (HCHO; 8.6 g·kg·d^−1^ CHO, 2.1 g·kg·d^−1^ protein, 1.2 g·kg·d^−1^ fat) or LCHF (0.5 g·kg·d^−1^ CHO, 2.1 g·kg·d^−1^ protein, 75–80% of energy from fat) diet followed by acute CHO restoration. Serum markers of bone breakdown (cross-linked C-terminal telopeptide of type I collagen, CTX), formation (procollagen 1 N-terminal propeptide, P1NP) and metabolism (osteocalcin, OC) were assessed at rest (fasting and 2 h post meal) and after exercise (0 and 3 h) at Baseline, after the 3.5-week intervention (Adaptation) and after acute CHO feeding (Restoration).

**Results:** After Adaptation, LCHF increased fasting CTX concentrations above Baseline (*p* = 0.007, Cohen's *d* = 0.69), while P1NP (*p* < 0.001, *d* = 0.99) and OC (*p* < 0.001, *d* = 1.39) levels decreased. Post-exercise, LCHF increased CTX concentrations above Baseline (*p* = 0.001, *d* = 1.67) and above HCHO (*p* < 0.001, *d* = 0.62), while P1NP (*p* < 0.001, *d* = 0.85) and OC concentrations decreased (*p* < 0.001, *d* = 0.99) during exercise. Exercise-related area under curve (AUC) for CTX was increased by LCHF after Adaptation (*p* = 0.001, *d* = 1.52), with decreases in P1NP (*p* < 0.001, *d* = 1.27) and OC (*p* < 0.001, *d* = 2.0). CHO restoration recovered post-exercise CTX and CTX exercise-related AUC, while concentrations and exercise-related AUC for P1NP and OC remained suppressed for LCHF (*p* = 1.000 compared to Adaptation).

**Conclusion:** Markers of bone modeling/remodeling were impaired after short-term LCHF diet, and only a marker of resorption recovered after acute CHO restoration. Long-term studies of the effects of LCHF on bone health are warranted.

## Introduction

Despite the generally positive effects of exercise in promoting bone health, bone injuries represent a challenge to consistent training and competition in high performance sport (1). This, in part, is due to the interaction of dietary factors (e.g., low energy availability, poor vitamin D status, inadequate calcium intake) with unique features of the exercise program [e.g., minimal or excessive bone loading associated with weight- and non-weight-bearing sports, poor biomechanics (1, 2)]. Low energy availability (a mismatch between energy intake and the energy cost of exercise) occurs in both female and male athletes (2) and impairs bone health via direct (uncoupled bone turnover with increased resorption rates) and indirect (mediation by reproductive and metabolic hormones) mechanisms (1). In addition, carbohydrate (CHO) availability may also play a role in bone health. Indeed, results from several studies show that commencing endurance exercise with low compared to normal or high glycogen availability stimulates the release of the cytokine interleukin-6 (IL-6) from the exercising muscles (3, 4). Among its range of effects, IL-6 has been hypothesized to lead to enhanced activity of the receptor activator of the nuclear factor K B-ligand, which controls bone turnover by increasing osteoclastic activity (thereby increasing bone breakdown) (5). In support of this contention, bone resorption is acutely increased when CHO is restricted before (6), during (7), and after (8) prolonged (1–2 h) endurance (running) exercise, and may be linked to concomitant increases in IL-6 concentrations (7). However, a recent study has reported that acute reductions in CHO availability around exercise mediated an increase in markers of bone resorption that are independent of energy availability and circulating IL-6 (9). Apparent effects on other markers of bone metabolism, such as osteocalcin (OC) and the bone formation marker procollagen 1 N-terminal propeptide (P1NP) in these models have been small (6–9), although a 24 h fast has been reported to reduce blood OC concentrations in lightweight rowers (10).

Whether these changes in markers of bone metabolism persist (or are amplified) after chronic exposure to low CHO availability around exercise remains unknown, but is of relevance in view of the promotion of a ketogenic low CHO-high fat (LCHF) diet to athletes and its putative benefits for endurance performance (11). To date, no studies have examined the effects of longer-term restriction of CHO at rest or in relation to exercise, although in animal models and children with intractable epilepsy, chronic adaptation to a ketogenic LCHF diet is associated with poor bone health (12–16). In view of our recent observations of increased post-exercise IL-6 concentrations in elite race walkers following a 3.5-week adaptation to a LCHF diet (17), we investigated the interaction of this diet and strenuous exercise on markers of bone modeling/remodeling as secondary outcomes of our larger study.

## Methods

### Participants

Thirty world-class athletes (25 male, 5 female race walkers; ages 27.7 ± 3.4 yr, BMI 20.6 ± 1.7 kg/m^2^) were recruited over three separate training camps during preparation for the 2016 Summer Olympic Games and the 2017 World Championships, and provided written informed consent in accordance with the Human Ethics Committee of the Australian Institute of Sport (ethics approval no. 20150802 and 20161201). Six male participants undertook two camps, however two of these data sets were incomplete due to insufficient tissue samples, resulting in 4 participants who had completed two camps being included in the final analysis. In addition, two additional (male) data sets were excluded from the final analysis due to their inability to complete one of the experimental trials due to injury (unrelated to bone). Therefore, our final data set provided a total of 32 trials (*n* = 28 participants, 23 males, 5 females) with data for pre- (*Baseline*) and post-treatment (*Adaptation*), of which 18 trials (13 males, 5 females) also contributed to data from acute restoration to a HCHO diet (*Restoration*). Participants and elite coaches contributed to the concept and implementation of the research camps, helping to prioritize the themes of interest and contributing to the design of the training program and test protocols.

### Study Overview

**Figure 1:**
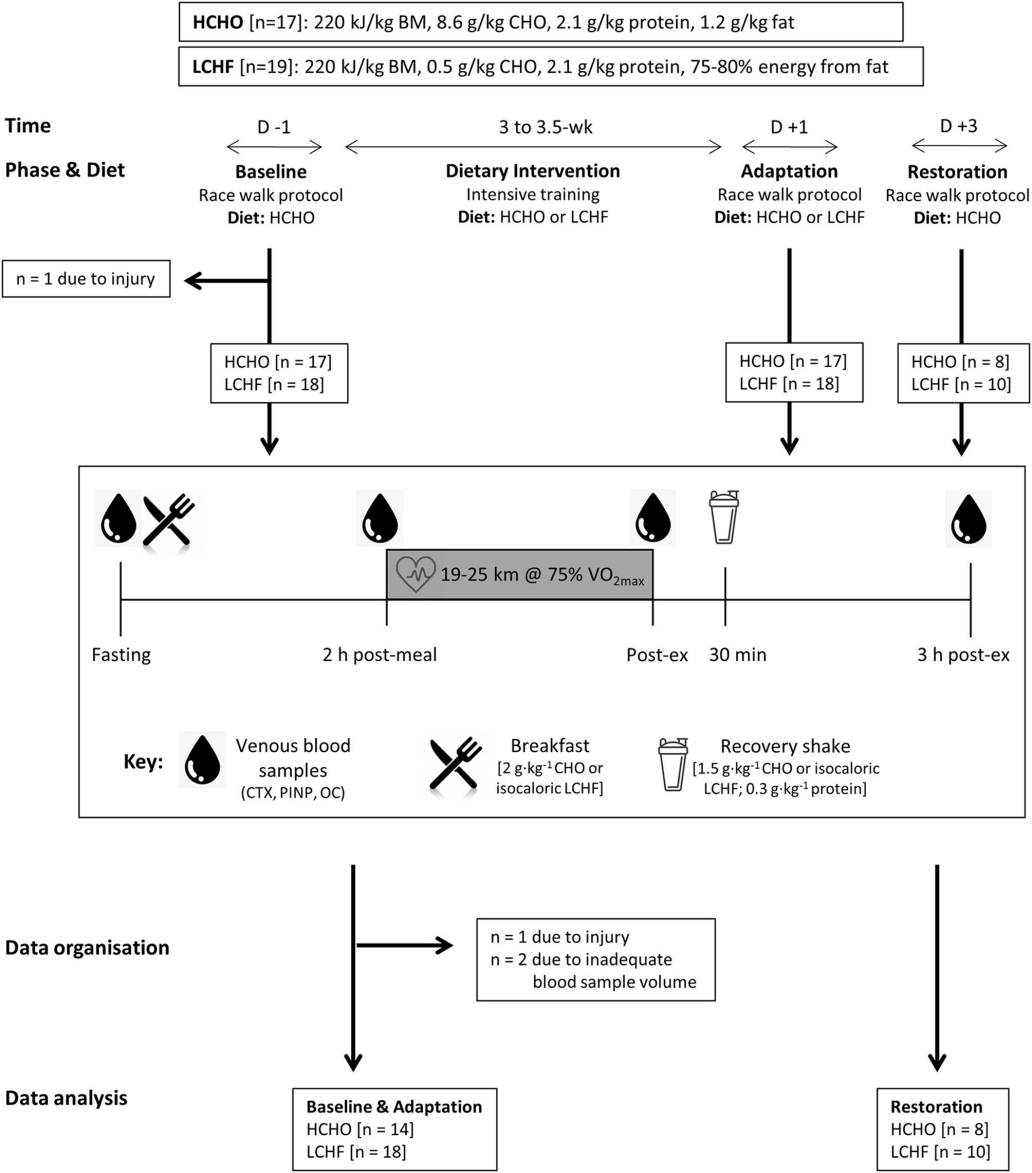
Study flowchart and overview. Thirty-two data sets were gathered from 30 participants who participated in one or more training camps. After Baseline testing on a carbohydrate-rich (HCHO) diet, they elected to follow a 3.5-week energy-matched dietary intervention of either HCHO or ketogenic low carbohydrate-high fat (LCHF) principles. After Adaptation, the participants underwent an acute period of Restoration of high carbohydrate availability. At Baseline and at the end (Adaptation) of this intervention, as well as after acute carbohydrate reintroduction (Restoration) they undertook a test block including a 25 km (2 h) hybrid laboratory/field race walking protocol at ~75% VO_2_ max. Venous blood samples were collected after an overnight fast, 2 h after an energy-matched breakfast based on their diet (immediately pre-exercise), immediately post exercise and after 3 h of passive recovery during which an intervention-matched recovery shake was consumed at 30 min. Blood samples were analyzed for serum concentrations of C-terminal telopeptide of type I collagen (CTX), procollagen 1 N-terminal propeptide (P1NP), and osteocalcin (OC).

Participants completed a 3.5-week block of intensified training and laboratory and field testing, supported by either a high-CHO (HCHO) or an isoenergetic LCHF diet (Figure 1, Table 1), consumed under strict dietary control (18). Upon completion of the 3.5-week dietary intervention, a subset of participants (*n* = 18) completed a further testing block under conditions of acute high CHO availability. Markers of bone metabolism were measured after an overnight fast, in response to an energy-matched meal of nutrient composition matching the intervention diet, and in response to a bout of strenuous exercise (19), at Baseline, Adaptation, and Restoration (Figure 1).

**Table 1:** Dietary intakes in the HCHO and LCHF groups.

	**Intervention**		**Restoration**	
	**HCHO (*n* = 14)**	**LCHF (*n* = 18)**	**HCHO (*n* = 8)**	**LCHF (*n* = 10)**
Energy (kJ· d^−1^)	14,518 ± 2,142	15,138 ± 2,104	13,705 ± 1,948	15,706 ± 1,774
Energy (kJ· kg· d^−1^)	229 ± 13	227 ± 23	219 ± 16	239 ± 27
Protein (g· d^−1^)	133 ± 22	143 ± 19	132 ± 24	151 ± 18
Protein (g· kg· d^−1^)	2.1 ± 0.2	2.1 ± 0.2	2.1 ± 0.2	2.3 ± 0.2
Fat (g· d^−1^)	74 ± 14	318 ± 45^***^	77 ± 14	95 ± 12^**^^$$$^
Fat (g· kg· d^−1^)	1.2 ± 0.1	4.8 ± 0.5^***^	1.2 ± 0.1	1.4 ± 0.2^**^^$$$^
CHO (g· d^−1^)	549 ± 75	35 ± 5^***^	492 ± 60^$^	552 ± 62^$$$^
CHO (g· kg· d^−1^)	8.7 ± 0.4	0.5 ± 0.1^***^	7.9 ± 0.6^$$^	8.4 ± 1.0^$$$^

*HCHO, high carbohydrate diet; LCHF, low carbohydrate high fat diet; CHO, carbohydrate*.

** *p < 0.01*,

*** *p < 0.001 significant difference between diets*.

$ *p < 0.05*,

$$ *p < 0.01*,

$$$ *p < 0.001 significantly different compared to Intervention*.

### Dietary Control

Details of dietary control are described briefly here; more details are described in prior work (18). Participants were allocated into HCHO and LCHF groups based on preference. Both diets were isocaloric (Table 1), however dietary CHO and fat intakes differed between groups during intervention. Study diets were designed and individualized for each athlete by trained members of the research team including registered sports dietitians, a professional chef, and exercise physiologists. All meals were weighed (food scales accurate to 2 g) and provided for athletes at set meal times. In addition, a collection of snacks per individual meal plans were provided to the athletes each day. Any unconsumed items or changes made to menu plans were weighed and recorded for final analysis of dietary intakes. Compliance to the meal plans was assessed daily. Meal plans were designed and final dietary analysis of actual intakes was conducted using FoodWorks 8 Professional Program (Xyris Software Australia Pty Ltd, Australia). Further analysis of intakes was completed using Microsoft Excel.

### Experimental Design

Testing at Baseline, Adaptation, and Restoration involved a hybrid laboratory/field test of 25 km (males) or 19 km (females) at around 50 km race pace (75% of maximal oxygen uptake [VO_2_ max]) (Figure 1). Upon entering the laboratory in an overnight fasted and rested state between 0600 and 0800 in the morning (times were kept consistent within-participant), a cannula was inserted into an antecubital vein for collection of blood samples at rest (Fasting), immediately before exercise (2 h post-meal), immediately after exercise (Post-ex) and 3 h post-exercise (3 h post-ex). Blood was analyzed for concentrations of cross-linked C-terminal telopeptide of type I collagen (CTX), P1NP and total OC to determine the effects of dietary interventions and exercise on bone metabolism. The cannulas were flushed with 3 ml of saline every 30 min throughout the trials. A standardized breakfast (2 g·kg^−1^ CHO for both groups during Baseline and Restoration, or an isocaloric low CHO option for LCHF during Adaptation) was consumed 30 min after the first blood sample, after which the participants rested for 120 min before beginning the session. During the Baseline and Restoration exercise test, both groups ingested glucose (60 g·h^−1^) throughout the test, while during Adaptation, isocaloric high fat snacks were provided for the LCHF group. Upon completion of the exercise test, the participants rested in the laboratory for a further 3 h, and received a standardized recovery shake (1.5 g·kg^−1^ CHO for both groups during Baseline and Restoration, or an isocaloric low CHO option for LCHF during Adaptation; both shakes included 0.3 g·kg^−1^ protein) at 30 min post-exercise to improve satiety.

### Analysis of Serum Bone Modeling/Remodeling Biomarkers

Blood samples were collected into a 3.5 mL EDTA BD Vacutainer Plus SST II tube, and allowed to clot by standing at room temperature for 2 h before centrifuging at 1,000 G for 10 min for subsequent analysis of serum markers of bone resorption (CTX), bone formation (P1NP) and overall bone metabolism (OC). Analysis was undertaken by chemiluminescence on IDS-iSYS (Immunodiagnostic Systems Limited; Boldon, Tyne and Wear, UK). Inter-assay coefficient of variation as reported by the manufacturer was 6.2, 4.6, and 6.1%, respectively. CVs were determined as follows: OC: 6 serum controls were run, using 3 reagents lots, in duplicate twice per day for 20 days, on 2 analyzers; P1NP: 3 serum controls were run, using 3 reagent lots, in quadruplicates once per day for 20 days, on 2 analyzers; CTX: 5 serum controls were run, using 3 reagent lots, in duplicate twice per day for 20 days, on 3 analyzers. In addition to these tests, the laboratory ran quality control samples throughout testing and the results were within the established acceptable manufacturer ranges. The raw data for the analyses of serum bone modeling/remodeling markers are provided in the Supplementary Table to this publication.

### Statistical Analyses

Statistical analyses were conducted using SPSS Statistics 22 software (INM, New York, USA) and R (R Core Team, 2018) with a significance level set at *p* ≤ 0.05. Normality of data was checked with a Shapiro-Wilk test and visual inspection of residual plots. General Linear Mixed models were fitted using the R package lme4 (20) and included random intercepts for Subjects and Camps to account for baseline inter individual heterogeneity and the partial cross-over design. Because the estimated Camp effect variance was 0, this random intercept was subsequently removed to resolve boundary issues in the Restricted Maximum Likelihood estimation. *P*-values were obtained using Type II Wald F tests with Kenward-Roger degrees of freedom. Initial models included all possible interactions but non-significant interaction terms were dropped for ease of interpretation. Fasting values and exercise-related area under curve [AUC; Pre-exercise to 3 h post-exercise (21)] for all markers were compared with a two-way mixed analysis of variance (ANOVA), with *post-hoc* tests of Student's *t*-tests for independent samples (between-groups) and for paired samples (within-groups); where normality was violated, Wilcoxon's test and Mann-Whitney U-test were used. Where a data point was missing, AUC was not calculated; this resulted in exclusion of 1 participant in the CTX AUC calculations, and 2 participants from both P1NP and OC calculations. Effect sizes were calculated based on the Classical Cohen's *d* while accounting for the study design by using the square root of the sum of all the variance components (specified random effects and residual error) in the denominator. Data are presented as means (95% confidence intervals [CI]).

## Results

### Bone Modeling/Remodeling Biomarkers During Fasting

**Figure 2:**
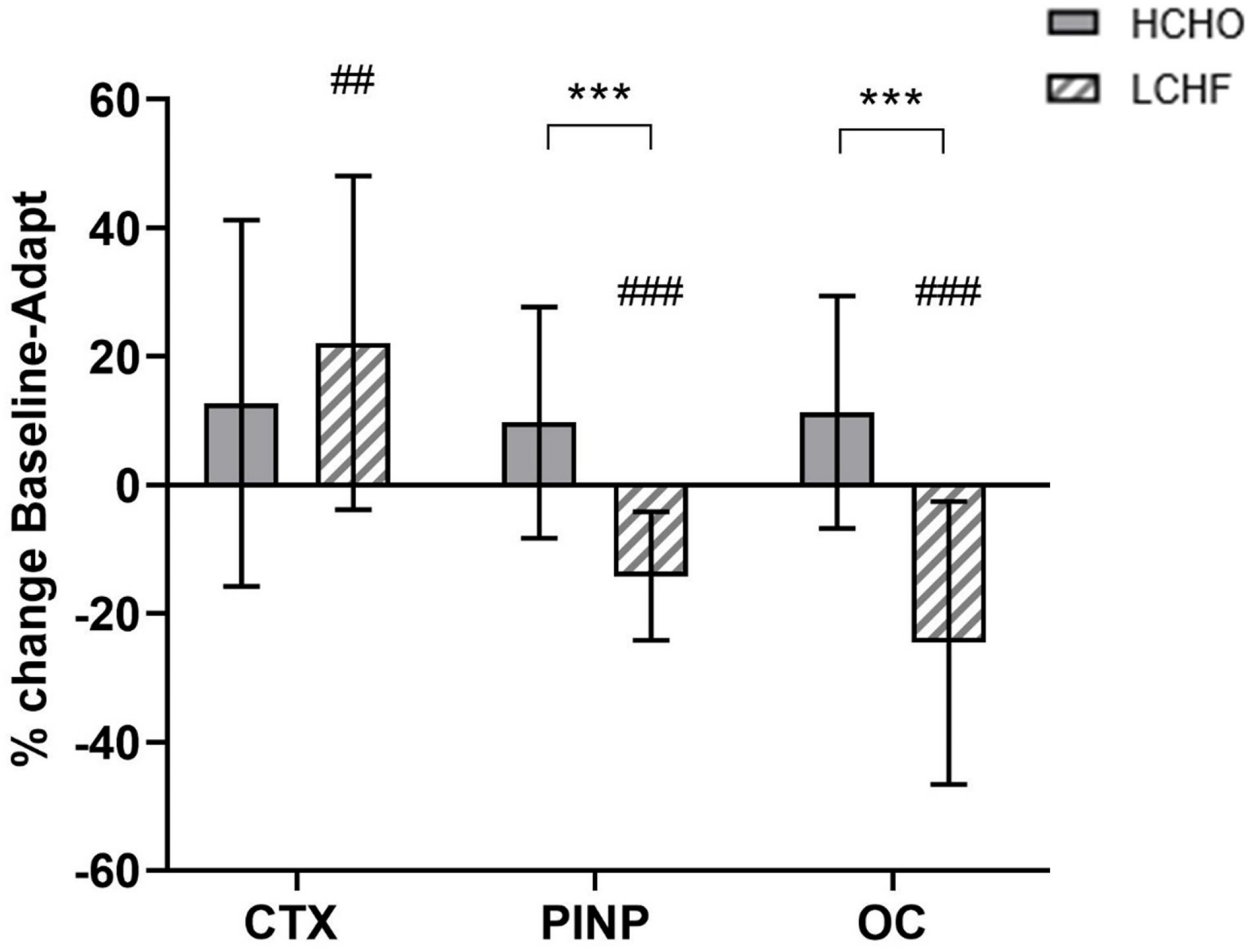
Percentage change in fasting serum C-terminal telopeptide of type I collagen (CTX), procollagen 1 N-terminal propeptide (P1NP) and osteocalcin (OC) for high carbohydrate (HCHO; solid bars) and low CHO high fat (LCHF; striped bars) after the 3.5-week dietary intervention. Data are means ± standard deviations. ***p < 0.001 Significant between-group difference; ^*##*^*p* < 0.01; ^*###*^*p* < 0.001 Significant change from Baseline within-group.

Compared to Baseline, fasting concentrations of CTX were increased after the LCHF diet (+22% [9, 35]: *p* = 0.008, *d* = 0.69), with a decrease in P1NP (−14% [−19, −9]; *p* = 0.001, *d* = 0.99) and OC (−25% [−35, −14]; *p* < 0.001, *d* = 1.39) levels (Figure 2). In addition, the change in fasting P1NP (*p* < 0.001, *d* = 1.64) and OC (*p* < 0.001, *d* = 1.78) after the 3.5-week intervention was significantly different between the diets (Figure 2).

### Exercise Bone Markers

**Figure 3:**
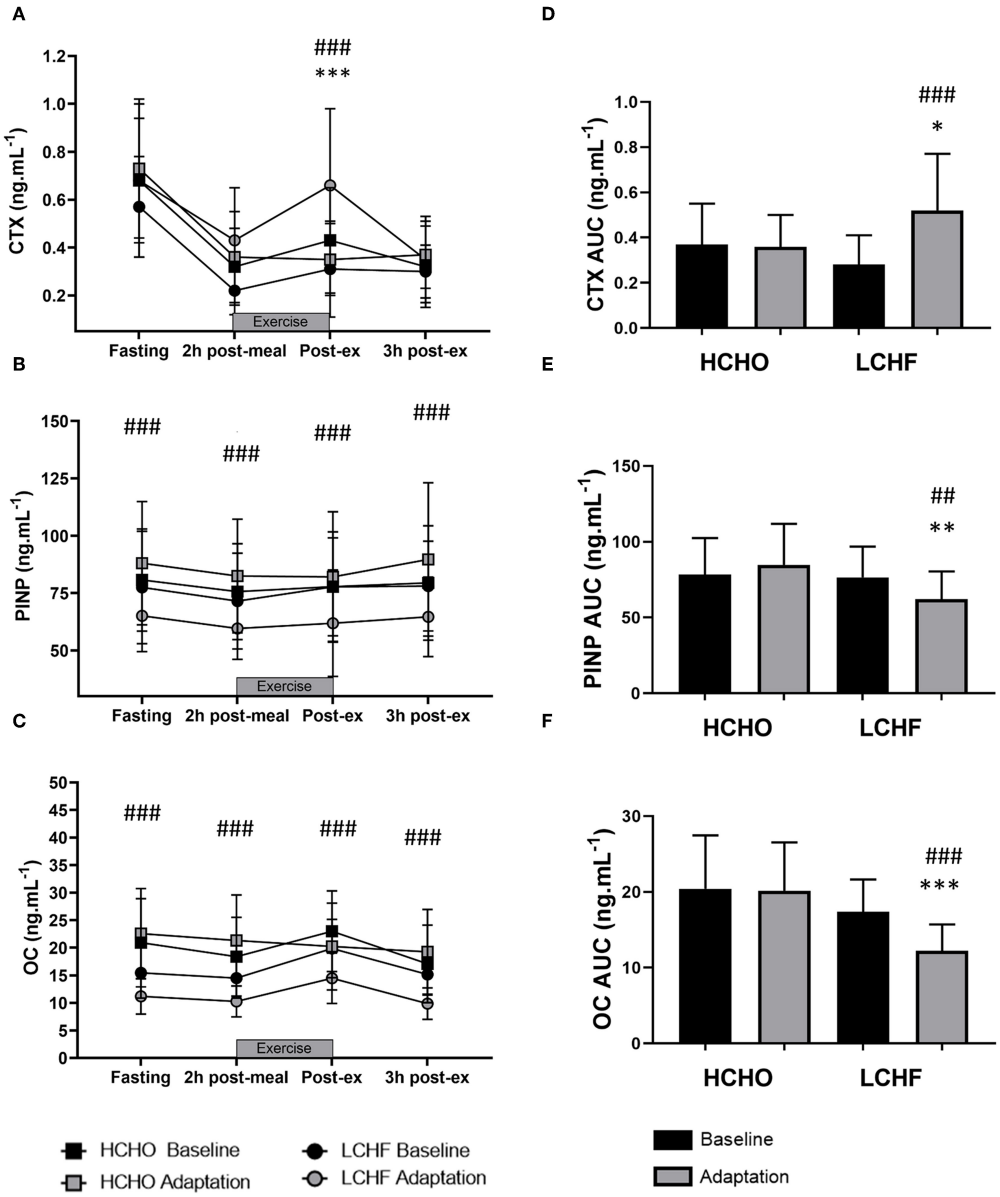
Time course of changes in bone marker concentrations across exercise (left panel) and exercise area under curve (right panel) for serum C-terminal telopeptide of type I collagen (CTX) **(A,D)**, procollagen 1 N-terminal propeptide (P1NP) **(B,E)**, and osteocalcin (OC) **(C,F)** after the 3.5-week dietary intervention. Black bars/symbols represent Baseline, gray bars/symbols represent Adaptation. Squares and circles represent high carbohydrate (HCHO) and low carbohydrate high fat (LCHF), respectively. Gray bars represent a hybrid laboratory/field 19–25 km walk test at ~75% VO_2_ max. Data are means ± standard deviations. ^*##*^*p* < 0.01; ^*###*^*p* < 0.001 denotes significant differences at time points or tests within diet groups. *p < 0.05; **p < 0.01; ***p < 0.001 denotes significant differences between diet groups at a specific time point.

**Figure 4:**
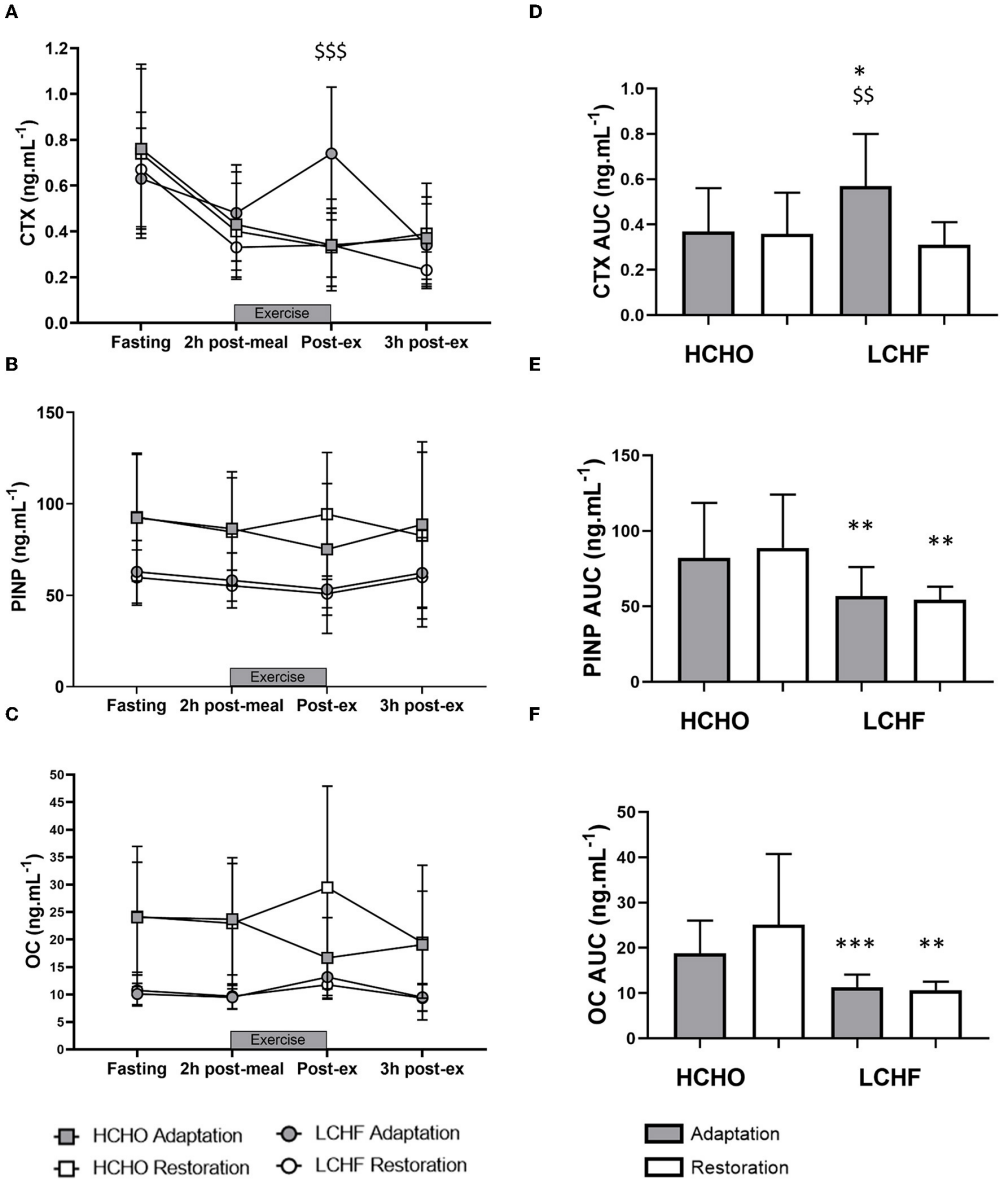
Time course of changes in bone marker concentrations across exercise (left panel) and exercise area under curve (right panel) for serum C-terminal telopeptide of type I collagen (CTX) **(A,D)**, procollagen 1 N-terminal propeptide (P1NP) **(B,E)**, and osteocalcin (OC) **(C,F)** after acute reintroduction of carbohydrate (right panel). Gray bars/symbols represent Adaptation, and white bars/symbols represent Restoration. Squares and circles represent high carbohydrate (HCHO) and low carbohydrate high fat (LCHF), respectively. Gray bars represent a hybrid laboratory/field 19–25 km walk test at ~75% VO_2_ max. Data are means ± standard deviations. ^$$^*p* < 0.01; ^$$$^*p* > 0.001 denotes significant within-group difference compared to Restoration. *p < 0.05; **p < 0.01; ***p < 0.001 denotes significant differences between diet groups at a specific time point.

CTX decreased post-meal independent of dietary intervention (Figures 3A, 4A, *p* < 0.001, *d* = 1.63). At Adaptation, post-exercise CTX concentrations in LCHF increased above Baseline (*p* = 0.001, *d* = 1.67) and HCHO (*p* < 0.001, *d* = 0.62) (Figure 3A). LCHF decreased P1NP (Figure 3B, *p* < 0.001, *d* = 0.85) and OC across exercise (Figure 3C, *p* < 0.001, *d* = 0.99) compared to Baseline. At Restoration, post-exercise CTX returned to Baseline levels for LCHF (Figure 4A, *p* > 0.05, *d* = 0.20 compared to Baseline), while concentrations of P1NP (Figure 4B, *p* < 0.001, *d* = 0.23) and OC (Figure 4C, *p* < 0.001, *d* = 0.21) remained suppressed across exercise.

### Bone Marker Exercise Area Under Curve

At Adaptation, LCHF exercise-related AUC for CTX was greater [+81% (54, 109); *p* < 0.001, *d* = 1.52] than Baseline, and higher than HCHO (*p* = 0.035, *d* = 0.81) (Figure 3D). Exercise-related AUC for P1NP decreased at Adaptation for LCHF [−19% (−25, −12); *p* = 0.003, *d* = 1.27] compared with Baseline and was lower than HCHO (*p* = 0.009, *d* = 1.03) (Figure 3E), with similar outcomes for OC [−29% (−35, −23); *p* < 0.001, *d* = 2.0 and *p* < 0.001, *d* = 1.64, Figure 3F]. At Restoration, LCHF experienced a return of exercise-related AUC for CTX back to Baseline values [−43% (−21, 31); *p* = 0.003, *d* = 1.08 compared to Adaptation and no difference compared to HCHO; Figure 4D], meanwhile AUC for P1NP [+3% (−17, 48), *p* = 1.000 compared to Adaptation and *p* = 0.009, *d* = 1.50 compared to HCHO; Figure 4E], OC [−3% (−19, 14), *p* = 1.000 compared to Adaptation and *p* = 0.010, *d* = 1.47 compared to HCHO; Figure 4F] remained suppressed.

## Discussion

Our data reveal novel and robust evidence of acute and likely negative effects on the bone modeling/remodeling process in elite athletes after a short-term ketogenic LCHF diet, including increased marker of resorption (at rest and post-exercise) and decreased formation (at rest and across exercise), with only partial recovery of these effects following acute restoration of CHO availability. Long-term effects of such alterations remain unknown, but may be detrimental to bone mineral density (BMD) and bone strength, with major consequences to health and performance. While ketogenic diets are of interest to athletes due to their ability to induce substantial shifts in substrate metabolism, increasing the contribution of fat-based fuels during exercise (11), we have previously reported the downside of a concomitantly greater oxygen cost and reduced performance of sustained high-intensity endurance exercise (19). The current study identifies further complexity in the interaction between the ketogenic diet and exercise with respect to markers of bone modeling/remodeling, in which catabolic processes are augmented and anabolic processes are reduced.

The LCHF diet is also popular within the general community for its purported health benefits, including rapid weight loss and improved glycemic control (22). However, data from animal studies (12, 13) demonstrate that chronic LCHF diets are associated with impaired bone growth, reduced bone mineral content, compromised mechanical properties, and slower fracture healing. Furthermore, increased bone loss has been reported in children with intractable epilepsy placed on a medically supervised LCHF diet for 6 months (14, 15). In contrast, adults with type 2 diabetes mellitus who self-selected to consume a LCHF diet for 2 years experienced no changes in spinal BMD in comparison to a “usual care” group (22). One explanation for these divergent outcomes involves interactions of the LCHF diet with the level of habitual contractile activity. Indeed in mice, a LCHF diet negated the positive benefits of exercise on BMD in trabecular bone (16), while in children with epilepsy, the rate of bone loss was greater in the more active patients (14). Therefore, the hormonal response to exercise undertaken with low CHO availability was of particular interest in our study.

Previous studies involving acute strategies of low CHO availability around exercise have identified effects on bone resorption, as measured by increased blood CTX concentrations. For example, males who undertook 60 min of treadmill running at 65% VO_2_ max following a CHO-rich breakfast (~1 g·kg^1^) showed small variations in CTX responses, but only around the exercise period, while dietary effects on parathyroid hormone, OC and P1NP were not detected (6). Meanwhile, a more strenuous protocol (120 min at 70% VO_2_ max) was associated with an attenuation of acute (pre-exercise to 2 h post-exercise) concentrations of IL-6, CTX, and P1NP when CHO was consumed (0.7 g·kg·h^1^) during exercise (7). However, OC was unchanged by diet and no differences in markers of bone metabolism were detected over the subsequent three days, suggesting that these effects are transient and quickly reversed (7). Short-term effects were also reported when 24 elite male runners with energy-matched intake over an 8 d period were divided into a group who consumed CHO before, during, and immediately after each of their 13 training sessions (additional total CHO) while the others consumed an artificially sweetened placebo (23). Here, CTX concentrations were suppressed at 80 min of recovery following an interval training sessions in the CHO group with no dietary effects on P1NP or OC; furthermore, fasting concentrations of all markers were similar at baseline and on the ninth morning (23). Finally, Hammond and colleagues (9) investigated the independent effects of low CHO availability and acute energy restriction during the recovery from one session of high-intensity interval running and the completion of a subsequent session (3.5 h into recovery). They reported lower CTX concentrations in the high CHO (control) diet compared with both of the other conditions across the various acute responses to exercise-related feeding, while there were no differences between the energy and CHO restricted trials. Meanwhile, only energy restriction produced an increase in IL-6 responses to exercise, and there were no differences in P1NP concentrations between dietary treatments (9). Furthermore, 5 d of low vs. optimal energy availability, which also resulted in a 2-fold difference in CHO availability, was shown to result in a significant difference in the AUC of fasting CTX (+85 vs. +15%, respectively) and P1NP (−60 vs. −25%, respectively) (24). To date, the only study to report an effect of acute manipulations of CHO around exercise on bone formation markers was that of Townsend et al. (8), in which the immediate consumption of a protein-CHO feeding after a run to exhaustion at 75% VO_2_ max was associated with a suppression of the post-exercise rise in CTX levels and a higher concentration of P1NP. These authors concluded that immediate post-exercise meal ingestion may benefit bone health compared to delayed feeding, although the effects on CTX concentrations were reversed at 4 h post-exercise and a similar time course of P1NP changes was not provided; therefore, it appears that the overall effect on bone modeling/remodeling processes appears to follow meal ingestion patterns.

The novelty of the current study was the interrogation of the effects of ***prolonged*** adaptation to CHO restriction on bone metabolism. Unlike the previous investigations, we identified clear and consistent effects on bone metabolism at rest and in response to exercise following 3.5-weeks of a ketogenic LCHF diet (Figures 2–4), with increases in a marker of bone resorption (CTX) and decreases in markers of bone formation (P1NP) and metabolism (OC). Although some might argue that a complete adaptation to a LCHF diet requires much longer than the 3.5-week period utilized in the current study, it should be noted that adaptations in substrate metabolism and exercise economy have been reported across this (19, 25), and much shorter (26), time periods. Nevertheless, the current study is reflective of a shorter-term adaptation to a LCHF diet and our findings warrant further investigation across longer time periods.

Acute restoration of high CHO availability was only partially effective in reversing these outcomes. Here, marker of bone resorption returned to baseline with high CHO pre-exercise meal and CHO ingestion throughout exercise, while the other markers of bone metabolism remained suppressed, indicating impaired overall balance of bone metabolism. This supports the concept proposed by Hammond et al. (9) that CTX is responsive to acute intake of CHO, possibly mediated through enteric hormone secretion. Meanwhile, differences in muscle glycogen content, which are not addressed by studies of acute feedings, may have a greater effect on OC and P1NP concentrations. Given the serious nature of injury risks and long-term outcomes of poor bone health in later life in endurance athletes, further consideration of the potential effects of the LCHF diet in exacerbating existing risk factors for poor bone health is warranted. In particular, we note that the impairment of bone metabolism around exercise and recovery would involve a significant portion of the day in athletes who undertake multiple training sessions, as well as being superimposed on the changes identified at rest.

The interaction of diet and exercise on bone metabolism is complex and requires more sophisticated investigation including replication of the current findings. Furthermore, evolving knowledge of inter-organ crosstalk suggests that outcomes of altered bone metabolism may be more far-reaching than the fate of the structural integrity of bone. Indeed, we note the recognition of muscle and bone as endocrine organs, with evidence that IL-6 released from contracting muscle has autocrine, paracrine and endocrine effects (27). This includes a purported feed-forward loop in which contraction-induced stimulation of osteocalcin in myofibers promotes the release of IL-6 and enhances muscle adaptation to exercise (27). Results of the current study challenge this synergistic relationship between osteocalcin signaling and IL-6, and remind us of the pleiotropic nature of the molecules stimulated by diet-exercise interactions.

### Limitations

The data analysis undertaken in this study was a secondary outcome of our investigations of the ketogenic LCHF diet; these were not specifically powered to optimally address the potential effects on markers of bone modeling/remodeling. However, the detection of changes in the IL-6 response to prolonged exercise in our initial study (12) provided motivation to examine possible downstream effects. Because an identical protocol was undertaken in two separate studies of the LCHF diet, we were able to pool data from these investigations to double the sample size previously known to allow detection of changes in metabolism and performance. Indeed, changes in markers of bone metabolism in the response to the interaction of exercise and the dietary treatments were clearly detected with the pooled data, but were also identifiable in the case of the smaller sample size of the carbohydrate restoration arm of the current dataset. Therefore, we feel confident that our data are robust and warrant further investigation of this theme.

## Conclusions

Despite recent interest in the potential benefits of LCHF diets on endurance performance or metabolic adaptation, the long-term health effects of this dietary intervention are largely unknown. We are the first to show that a 3.5-week ketogenic LCHF diet in elite endurance athletes has negative effects on the markers of bone modeling/remodeling at rest and during a prolonged high intensity exercise session. We also show only partial recovery of these adaptations with acute restoration of CHO availability. Given the injury risks and long-term outcomes underpinned by poor bone health in later life, in athletes as well as individuals who undertake exercise for health benefits, additional investigations of the ketogenic diet and its role in perturbing bone metabolism are warranted.

## Data Availability Statement

The datasets analyzed for this study were harvested from 2 trials registered at Australian New Zealand Clinical Trial Registry (ACTRN12619001015134 and ACTRN12619000794101), found at: http://www.ANZCTR.org.au/ACTRN12619001015134.aspx and http://www.ANZCTR.org.au/ACTRN12619000794101.aspx.

## Ethics Statement

The studies involving human participants were reviewed and approved by Australian Institute of Sport Ethics Committee. The patients/participants provided their written informed consent to participate in this study.

## Author Contributions

Conception and design of the experiments was undertaken by IH, LB, MR, LG-L, AS, AM, JL, MW, LM, and KA. Collection, assembly, analysis, and interpretation of data was undertaken by IH, LB, MR, LG-L, AS, AM, JL, MW, LM, and KA. Manuscript was prepared by IH, LB, KA, and JH. All authors approved the final version of the manuscript. IH and LB had full access to all the data in the study and take responsibility for the integrity of the data and the accuracy of the data analysis.

### Conflict of Interest

The authors declare that the research was conducted in the absence of any commercial or financial relationships that could be construed as a potential conflict of interest.

## Supplementary Material

Supplementary Table 1 Individual data for the serum concentrations of bone modeling/remodeling markers (CTX, P1NP and Osteocalcin) in response to strenuous exercise (2 h race walking) and dietary interventions (high carbohydrate and low carbohydrate high fat diets) in elite race walkers.
